# Gangliosides in the 21st century: therapeutic prospects for the brain and spine

**DOI:** 10.3389/fneur.2026.1795901

**Published:** 2026-06-15

**Authors:** Pierre J. Magistretti, Charles Finsterwald, Yutaka Itokazu, Simonetta Sipione, Fred H. Geisler, Tarcisio Barros Filho

**Affiliations:** 1 Biological and Environmental Science and Engineering Division, King Abdullah University of Science and Technology, Thuwal, Saudi Arabia; 2 GliaPharm, Geneva, Switzerland; 3 Department of Pharmacology & Toxicology, Medical College of Georgia, Augusta University, Augusta, GA, United States; 4 Department of Neuroscience and Regenerative Medicine, Medical College of Georgia, Augusta University, Augusta, GA, United States; 5 Department of Pharmacology, Neuroscience and Mental Health Institute, Glycomics Institute of Alberta, University of Alberta, Edmonton, AB, Canada; 6 Department of Small Animal Clinical Sciences, Western College of Veterinary Medicine, University of Saskatchewan, Saskatoon, SK, Canada; 7 Department of Orthopaedics and Traumatology, Faculty of Medicine, University of São Paulo, São Paulo, Brazil

**Keywords:** acute spinal cord injury (ASCI), astrocyte, GM1, Huntington (disease), neurodegenenerative diseases, microglia, extracellular vesicles

## Abstract

Gangliosides are sialylated glycosphingolipids highly enriched in the central nervous system, where they regulate membrane signaling, metabolism, neurogenesis, and immune responses. This Review integrates recent advances across distinct experimental and clinical domains. First, recent studies demonstrate that the monosialoganglioside GM1 enhances astrocyte–neuron metabolic coupling via the astrocyte–neuron lactate shuttle, thereby supporting neuronal bioenergetics and resilience. Complementary mechanistic work shows that specific gangliosides regulate adult neurogenesis through developmentally controlled epigenetic and transcriptional programs. In Huntington’s disease models, preclinical evidence indicates that GM1 and related gangliosides attenuate microglia-mediated inflammatory responses and promote proteostasis through extracellular vesicle–dependent clearance of misfolded proteins. Finally, clinical evidence from acute spinal cord injury demonstrates that GM1 administration accelerates neurological recovery, underscoring its translational relevance. Together, these findings position gangliosides as multi-target modulators of neural repair and inflammation, and highlight their potential for therapeutic development.

## Introduction

Gangliosides are specialized glycosphingolipids integral to the structure and function of cellular membranes, prominently in the central nervous system (CNS). Structurally, gangliosides comprise a ceramide lipid core linked to an oligosaccharide chain characterized by one or several sialic acid (N-acetylneuraminic acid) residues. The remarkable diversity among gangliosides arises from variations in the oligosaccharide chains, with over 200 distinct natural gangliosides identified thus far. Within the CNS, gangliosides represent approximately 10% of total lipid content, predominantly localized in neuronal and glial cell membranes, underscoring their crucial biological role.

Aberrations in ganglioside metabolism can significantly impact neurological function. Deficiencies in specific enzymes involved in ganglioside biosynthesis and degradation result in gangliosidoses, a group of inherited metabolic disorders characterized by severe neurological impairment due to ganglioside accumulation. Conversely, decreased ganglioside levels have been observed in several neurodegenerative diseases, including Alzheimer disease (AD), Parkinson disease (PD), and Huntington disease (HD), suggesting their potential therapeutic relevance (1–3). This bidirectional implication highlights the critical balance required for ganglioside homeostasis and normal CNS function (4, 5).

Among various gangliosides, GM1 has garnered considerable attention due to its demonstrated neuroprotective and neurorestorative properties in diverse preclinical and clinical studies. GM1 exerts its effects via multiple mechanisms, including modulation of cell signaling pathways, enhancement of neurogenesis, and protection against inflammatory damage and oxidative stress. Notably, GM1’s role in facilitating neuron–glia interactions, especially through the astrocyte-neuron lactate shuttle (ANLS), underscores its therapeutic potential (6–8). Astrocytes, the predominant glial cell type in the CNS, provide metabolic, structural, and neuroprotective support, influencing synaptic plasticity through gliotransmission (9, 10). Within the ANLS framework, glucose is metabolized into lactate by astrocytes and subsequently supplied to neurons as an essential energy substrate, especially during heightened neuronal activity and memory consolidation processes (7, 11).

Recent evidence demonstrates GM1’s potent influence on astrocyte-neuron metabolic interactions. GM1 enhances glucose uptake, glycogen breakdown, and lactate production in astrocytes, thereby strengthening the ANLS (6). Importantly, GM1-mediated improvements in neuronal mitochondrial function and neuroprotective gene expression rely on astrocyte-neuron interactions, indicating a critical synergy between these cell types. Such interactions are mediated by the mitogen-activated protein kinase (MAPK) signaling pathway, which is robustly activated by GM1 in neurons, provided astrocytes are present (4). This coordinated action suggests GM1 may specifically bolster neuronal resilience by reinforcing astrocytic metabolic support.

Beyond metabolic regulation, gangliosides play pivotal roles in neurogenesis, essential for maintaining the brain’s plasticity and repair mechanisms. Ganglioside species vary markedly across developmental stages, reflecting differential expression patterns of ganglioside synthase enzymes regulated partly through epigenetic mechanisms (12). During embryonic neurodevelopment, specific gangliosides like GD3 facilitate neural stem cell (NSC) proliferation (13). Postnatally, GD3 remains critical for sustaining NSC populations and modulating neurogenesis and synaptic development (13, 14). Loss of GD3 synthesis disrupts neuronal connectivity, leading to functional deficits, including impaired memory and depressive behaviors, underscoring ganglioside regulation’s broad neurological implications (15).

In contrast, GM1 and related brain-type gangliosides promote terminal neuronal differentiation through intricate interactions with chromatin. GM1 facilitates neuronal differentiation by recruiting transcription factors to gene promoters, enhancing gene expression profiles favoring neuronal maturity (16, 17). Such nuclear interactions underline GM1’s dual functional roles—both as a membrane constituent modulating signaling cascades and a nuclear regulator influencing gene transcription.

Therapeutically, gangliosides have been examined extensively in neurodegenerative models. In Alzheimer and Parkinson disease models, reduced brain ganglioside levels correlate with disease progression, while exogenous administration of GM1 or GD3 improves neuronal survival and functional outcomes (1, 18). GM1 administration significantly ameliorates neuropathological features, such as amyloid-*β* aggregation in AD models and *α*-synuclein aggregation in PD models (5, 19). Intriguingly, combined administration of GM1 and GD3 markedly boosts neurogenesis, rescues neuronal populations, and restores behavioral deficits, suggesting synergistic therapeutic effects (14, 20).

The utility of gangliosides is also promising in the context of HD, a hereditary neurodegenerative disorder associated with mutant huntingtin (mHTT) aggregation. GM1 administration in HD models halts or slows down neurodegeneration, restores normal motor and non-motor functions in mice, and effectively reduces mHTT accumulation, at least in part through enhanced extracellular vesicle (EV)-mediated clearance mechanisms (21–23). Moreover, GM1 administration attenuates microglial activation and inflammatory responses (24, 25).

Furthermore, clinical studies have validated GM1’s therapeutic efficacy in acute neurological injuries, particularly spinal cord injury (SCI). The extensive multicenter Sygen trial demonstrated GM1’s ability to accelerate neurological recovery significantly (26). Patients receiving GM1 exhibited faster improvements in motor and sensory functions, particularly when treatment was combined with early surgical intervention. These clinical benefits likely stem from GM1’s pleiotropic effects—stabilizing neuronal membranes, modulating calcium influx, promoting neurotrophic signaling, and enhancing reparative microglial responses (4, 26).

Thus, accumulating evidence underscores the therapeutic potential of gangliosides, particularly GM1, in addressing diverse neurological disorders. Their multifaceted mechanisms—ranging from enhancing neuronal metabolism and differentiation to modulating inflammatory and neurodegenerative processes—highlight their broad applicability and effectiveness. Nonetheless, further research is essential to optimize therapeutic regimens, elucidate precise molecular mechanisms, and define specific patient populations likely to benefit from ganglioside-based therapies. This topical review explores recent advancements in understanding ganglioside biology, their molecular targets, mechanisms of action, and clinical applications. By synthesizing current knowledge, we aim to outline the therapeutic landscape and identify critical future directions to harness gangliosides’ full clinical potential in neurological medicine.

## Gangliosides and energy metabolism: GM1 acts on the astrocyte neuron lactate shuttle (ANLS)

Astrocytes are the most abundant subtype of glial cells in the human CNS. Although they do not directly transmit electrical signals, astrocytes are essential for a wide range of metabolic, structural, homeostatic, and neuroprotective functions (8, 27). They provide critical energetic and structural support to neurons and regulate the clearance and recycling of neurotransmitters such as glutamate and gamma-amino butyric acid (GABA). More recently, astrocytes have been shown to influence neuronal plasticity and modulate extracellular ion concentrations, contributing to the fine-tuning of synaptic activity (9, 10). These roles collectively constitute the basis of gliotransmission, a process increasingly recognized for its importance in synaptic modulation.

**Figure 1:**
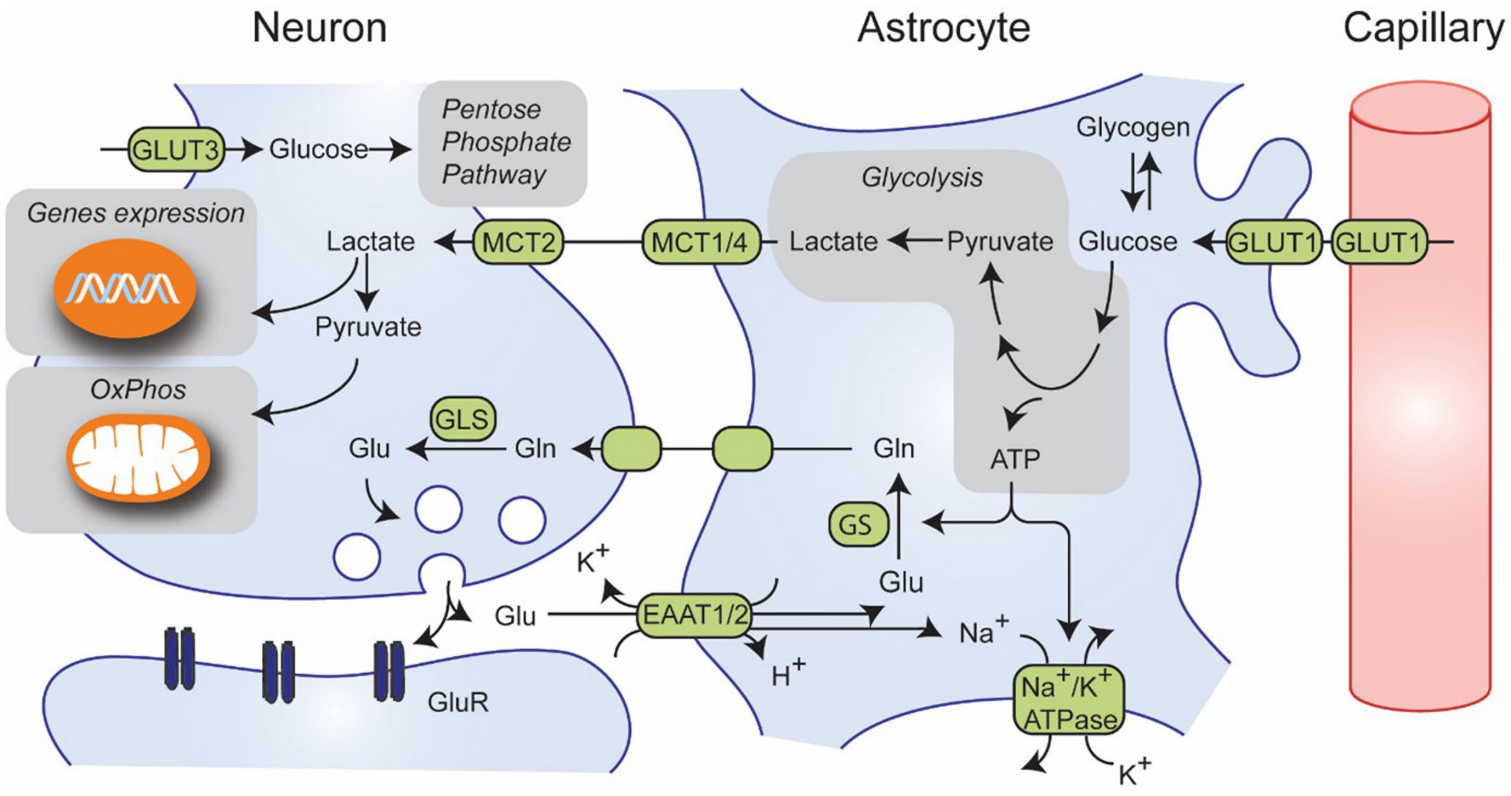
The astrocyte-neuron lactate shuttle. Aerobic glycolysis in astrocytes is driven by glucose uptake from capillaries via glucose transporter 1 (GLUT1) and by the mobilization of glycogen, the storage form of glucose in astrocytes. Glycolysis in astrocytes results in the production of lactate, which is exported through monocarboxylate transporters 1 and 4 (MCT1/4), and taken up by neurons via monocarboxylate transporter 2 (MCT2). In neurons, lactate serves as a signaling molecule that promotes the expression of synaptic plasticity and neuroprotection genes, as well as an energy substrate for oxidative phosphorylation (OxPhos) upon its conversion into pyruvate and entry into the tricarboxylic acid (TCA) cycle. While neurons can also import glucose directly via glucose transporter 3 (GLUT3), this pathway primarily supports the pentose phosphate pathway, which maintains redox homeostasis and the antioxidant defense system in neurons. The glutamate-glutamine cycle plays a critical role in neurotransmitter recycling and coupling to energy supply. During synaptic activity, glutamate is taken up from the synaptic cleft into astrocytes via excitatory amino acid 1 and 2 transporters (EAAT1/2). In astrocytes, glutamate is converted into glutamine by glutamine synthetase (GS). Glutamine is then shuttled back to neurons, where it is converted into glutamate by glutaminase (GLS) to replenish vesicles’ stores. Astrocytic energy demand rises during glutamate uptake due to the activation of the Na^+^/K^+^ ATPase to restore Na^+^ and K^+^ ion gradients following co-transport of glutamate through EAAT1/2, as well as glutamate to glutamine conversion by GS. These energy requirements are met by increased astrocytic glycolysis, thereby providing a mechanistic link between neuronal activity and energy supply via lactate-mediated support from astrocytes.

The brain’s high energy demands depend primarily on glucose metabolism. Astrocytes and neurons exhibit distinct metabolic profiles - astrocytes are primarily glycolytic, while neurons produce energy mainly through mitochondrial oxidative phosphorylation (7). Glucose, the main energy substrate of the brain, enters the brain parenchyma predominantly via astrocytes, where it is metabolized via glycolysis to produce lactate. Lactate is subsequently exported to neurons via the ANLS (Figure 1). The uptake of glutamate by astrocytes during synaptic activity leads to an increase in energy requirements in astrocytes, as ATP is needed to activate the Na^+^/K^+^ ATPase in order to restore ion gradients, and glutamine synthetase (GS) that converts glutamate into glutamine and is central to the glutamate-glutamine recycling cycle (Figure 1). This heightened energy requirement in astrocytes upon glutamate uptake results in the stimulation of aerobic glycolysis to produce ATP, leading to enhanced glucose uptake, glycogen mobilization and lactate release. This neurometabolic coupling provides a direct mechanistic link between neuronal activity and transfer of energy from astrocytes to neurons, which forms the basis of the ANLS (8, 27).

Lactate is not only a metabolic byproduct that fuels the tricarboxylic acid (TCA) cycle in neurons’ mitochondria to generate ATP through oxidative phosphorylation, but is also a dynamic signaling molecule that plays a central role in synaptic plasticity, neuroprotection, and cognitive functions. Following its release from astrocytes, lactate activates neuronal signaling cascades that regulate the expression of multiple genes involved in synaptic plasticity and neuroprotection, such as Activity-regulated cytoskeleton-associated protein (*Arc*), Early growth response 1 (*Egr1*) and Brain-derived neurotrophic factor (*Bdnf*) (28, 29). Consequently, the transfer of lactate from astrocytes to neurons via the ANLS has been identified as crucial for physiological processes like synaptic plasticity and the consolidation of long-term memory (11). Beyond synaptic plasticity and memory consolidation, lactate also mediates neuroprotection in ischemic, hypoxic, and traumatic contexts, reduces oxidative stress, and supports adult neurogenesis (30–32). Impairments in astrocytic glycolysis and lactate dynamics contribute to cerebral hypometabolism, a hallmark of aging and neurodegenerative diseases such as AD, PD, amyotrophic lateral sclerosis (ALS) and HD (33, 34).

Gangliosides exert wide-ranging effects on both neurons and glial cells and have long been known to influence neuroplasticity and neuroprotection (35–37). Among the different types of gangliosides, GM1 has shown particular promise in modulating neuroprotective and neurometabolic functions. Beyond its established effects on neuronal membrane signaling, recent findings underscore its capacity to modulate astrocytes’ physiology. First, GM1 was shown to promote the astrocyte-dependent activation of the MAPK signaling pathway in neurons (4). Additionally, a recent study from our group demonstrated that GM1 stimulates glycolysis in astrocytes derived from primary mouse brain cultures, promoting increased glucose uptake, transient glycogen mobilization, and elevated lactate secretion (6). GM1 upregulated several key metabolic genes in astrocytes, including glucose transporter 1 (*Glut1*), protein targeting to glycogen (*Ptg*), Hexokinase 2 (*Hk2*), pyruvate dehydrogenase (*Pdh*) and Lactate dehydrogenase A (*LdhA*), reflecting robust activation of glycolysis. In astrocyte-neuron co-culture system, GM1 markedly enhanced neuronal mitochondrial activity, while it did not have an impact on mitochondrial function in isolated astrocyte or neuron cultures, underscoring the importance of astrocytes to promote the metabolic effects of GM1 on neurons. In addition, GM1 was shown to increase NADH/NAD^+^ redox ratio in neurons in co-cultures, and to upregulate the expression of synaptic and neuroprotective genes such as *Arc*, Early growth response 4 (*Egr4*), and Nuclear receptor subfamily 4 group A member 3 (*Nr4a3*) (6). Importantly, our study also demonstrated that GM1 conferred neuroprotection against glutamate-induced excitotoxicity, but only in the presence of astrocytes, underscoring once more the indispensable role of astrocyte-neuron metabolic coupling in mediating GM1’s neuroprotective actions. These findings indicate that at least some of the beneficial effects of GM1 arise not from direct neuronal stimulation, but rather from its modulation of astrocytic glycolytic output and lactate transfer through the ANLS (6).

Together, these observations position GM1 as a promising therapeutic candidate for neurodegenerative diseases characterized by brain energy deficits. Its ability to potentiate astrocytic energy metabolism and enhance neuron viability through lactate signaling offers a mechanistic explanation for previously observed neuroprotective outcomes in neurological conditions such as PD, HD, and SCI.

## Gangliosides regulate neurogenesis: relevance for neurodegenerative disease treatments

Gangliosides undergo dynamic qualitative and quantitative changes related to development, age, cell specificity, and pathology, all of which correlate with neuronal function. Gangliosides play a crucial role during neurogenesis, i.e., for NSC quiescence, self-renewal, proliferation, differentiation, interaction, migration, and signal transduction during embryonic nervous system development and in the postnatal brain. Specific ganglioside species contribute to the differentiation of NSC into various neural cell types, such as astrocytes, oligodendrocytes, and neurons (38). Depending on the developmental stage, the tissue concentrations of various ganglioside species vary. In mice, GD3 displays maximum expression on embryonic days 12–14. Expression of GM1, GD1a, GD1b, and GT1b increase during neuronal differentiation, and those gangliosides became the major brain-type gangliosides in the postnatal brain (12).

**Figure 2:**
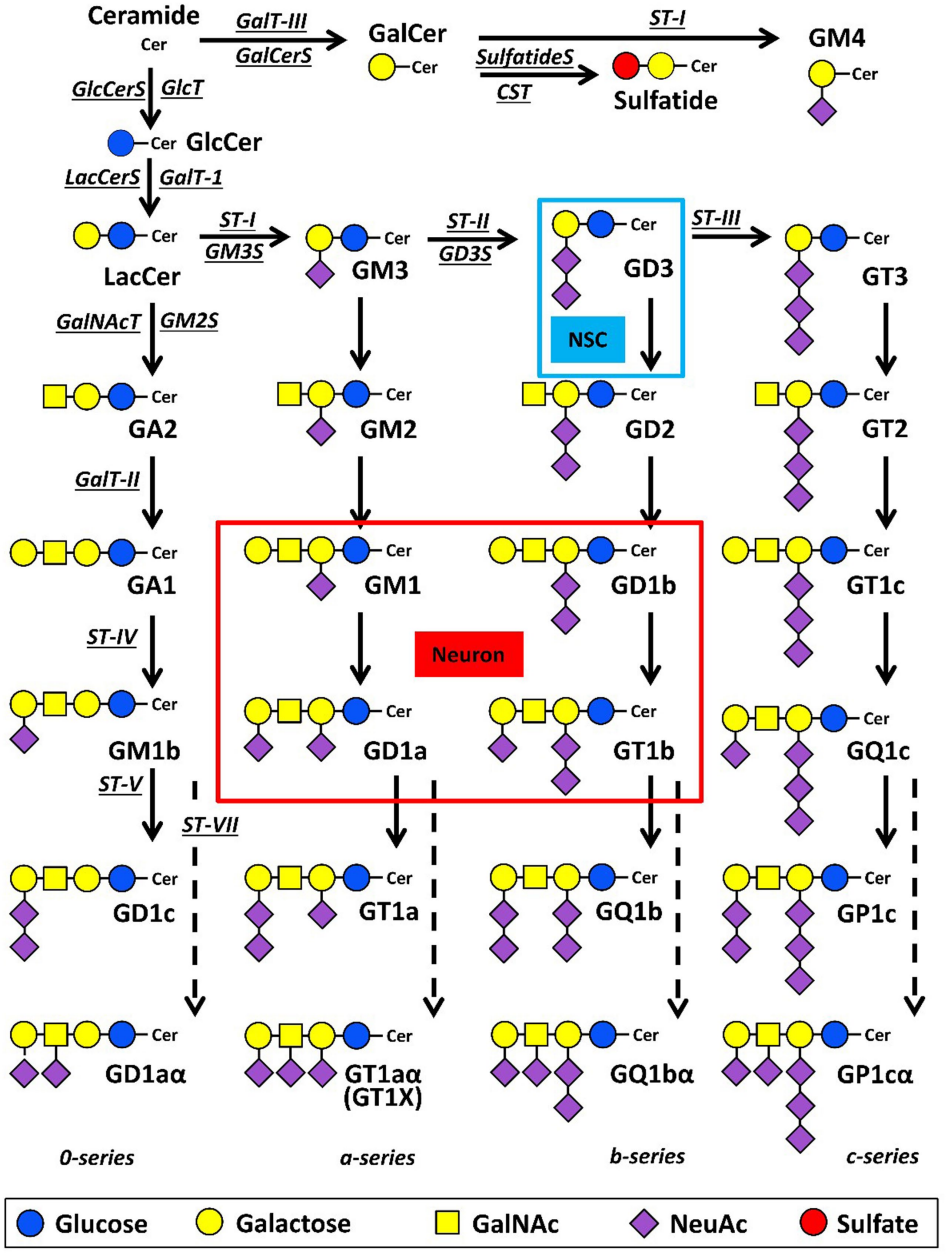
Metabolic pathways and structure of gangliosides. Cer ceramide, CST cerebroside sulfotransferase (*Gal3st1*, sulfatide synthase), GalNAcT *N*-acetylgalactosaminyltransferase I (*B4galnt1*, GA2/GM2/GD2/GT2 synthase), GalT-I galactosyltransferase I (*B4galT5* & *B4galt6*, lactosylceramide synthase), GalT-II galactosyltransferase II (*B3galt4*, GA1/GM1/GD1b/GT1c synthase), GalT-III galactosyltransferase III (*Ugt8a*, galactosylceramide synthase), GlcT glucosyltransferase (*Ugcg*, glucosylceramide synthase), ST-I sialyltransferase I (*St3gal5*, GM3 synthase), ST-II sialyltransferase II (*St8Sia1*, GD3 synthase), ST-III sialyltransferase III (*St8Sia3*, GT3 synthase), ST-IV sialyltransferase IV (*St3gal2*, GM1b/GD1a/GT1b/GQ1c synthase), ST-V sialyltransferase V (*St8sia5*, GD1c/GT1a/GQ1b/GP1c synthase), ST-VII sialyltransferase VII (*St6galnac6*, GD1aα/GT1aα/GQ1bα/GP1cα synthase). Official symbols of genes are represented in italics.

The alterations in ganglioside concentrations over time are mainly due to stage-specific expression patterns of the enzymes involved in their synthesis, i.e., specific glycosyltransferases also called ganglioside synthases. Ganglioside synthesis pathways were initially delineated by Yu and Ando (Figure 2) (39). These expression patterns appear to be at least in part under epigenetic control (40). In mice, histone H3 and H4 acetylation were shown to increase on the promoter of the genes encoding N-acetyl-galactosaminyl-transferase I (GalNAcT, GA2/ GM2/ GD2/ GT2 synthase; *B4galnt1*) and, those acetyl modifications were also increased on the *Sialyl-transferase II (GD3 synthase; St8sia1)* promoter, albeit at a much smaller rate of increments (17, 41). In humans, hereditary ganglioside deficiencies due to GM2 and GM3 synthase mutations are known to result in various neurological syndromes and symptoms. In mice, knock-out mutations of glucosylceramide (GlcCer) and lactosylceramide (LacCer) synthases turned out to be lethal, those synthases catalyzing subsequent metabolic transformations to result in major neurological defects (42).

GD3 is the predominant ganglioside species in NSCs maintaining their stemness (12). After birth, GD3 is required for long-term maintenance of NSCs in the brain (13–15). Deletion of GD3 not only impairs neurotrophin-induced NSC self-renewal, but also alters the dendritic structure as well as the number of synapses of nascent neurons in the dentate gyrus of adult brain (15). NSC specific GD3-synthase knockout mice displayed reduction of postnatal NSC pools in both the subventricular zone (SVZ) and the dentate gyrus in the hippocampus with severe developmental and behavioral deficits such as depression, olfactory dysfunction, and impairment of hippocampus-dependent memory function (13, 15, 43). Exogenous administration of GD3 significantly restored the NSC pools and enhanced the stemness of NSCs, i.e., multipotency and the capability of self-renewal (16, 20, 43). By contrast, brain-type gangliosides, i.e., GM1, GD1a, GD1b, and GT1b, resulted in terminal differentiation and loss of stemness of NSCs (12).

Regarding their mode of action, GD3 was shown to sustain EGFR signaling, maintain mitochondrial dynamics during neurogenesis (15, 16, 43) through binding of Dynamin-related protein-1 (DRP1) (15, 16), and modulate expression of cyclin-dependent kinase (CDK) inhibitors p27 and p21 (14, 43, 44). GM1 interacts with active chromatin via acetylated histones to recruit transcription factors on the neuronal gene promoters, resulting in gene expression toward neuronal differentiation (16). Nuclear GM1 was shown to bind acetylated histones at the promoters of *GM2 Synthase* and *NeuroD1* genes in differentiated neurons (16). Histone acetylation at the *GM2 Synthase* gene promoter resulted in recruitment of trans-activation factors Sp1 and AP-2 which triggered neuronal differentiation of NSCs in response to exogenous GM1 (16, 17, 45). GM1 can induce activation of the *Tyrosine Hydroxylase (TH)* gene by recruitment of Nuclear receptor related 1 (NURR1), a dopaminergic neuron-associated transcription factor also known as NR4A2 (16) on the *TH* promoter.

Neurodegenerative diseases such as AD and PD are associated with reduced complex ganglioside expression (3, 18, 42), and lower concentrations of these gangliosides have been found in the frontal brain areas of patients with AD (1). Yet, there is no definitive proven disease-modifying therapy for patients with neurodegenerative diseases. In Jay Schneider et al.’s long-term NIH-funded research (5), bovine-derived GM1 has been extensively studied in many PD patients over many years. The safety profile of GM1 trials was very impressive, and the improvements in movement disorders, along with the potential beneficial mechanisms of action of GM1 in PD, are encouraging. Concerns arose about the source of GM1 due to bovine spongiform encephalopathy (BSE), and efficient routes of administering GM1 into the brain have not yet been settled.

Intracerebroventricular infusion of GD3 increased the number of NSCs in GD3-synthase knockout mice and GD3 and GM1 synergistically promoted neurogenesis in the 5xFAD mouse model (20). Lewy bodies predominantly composed of *α*-synuclein (αSyn) are considered a hallmark of PD which concur with a loss of pigmented midbrain dopaminergic neurons. Intranasal infusion of GD3 and GM1 to the A53T mice, an αSyn overexpressing PD model, reduced intracellular αSyn concentrations (19, 42). GM1 also significantly enhanced expression of TH in the *substantia nigra pars compacta* and striatum, following restoration of nuclear expression of Nurr1 transcription factor. While neurodegeneration of dopaminergic neurons in the substantia nigra is mainly associated with motor symptoms of PD, non-motor symptoms such as hyposmia, sleep disturbances, depression, anxiety, and cognitive impairment are rather due to olfactory bulb and hippocampus dysfunctions and were suggested to be linked to adult neurogenesis. Intranasal GD3 administration rescued the number of bromodeoxyuridine (BrdU+)/Sox2 + NSCs in the SVZ (43). GD3 and GM1 together increased doublecortin-expressing immature neurons in the olfactory bulb and recovered the neuronal populations in the peri-glomerular layer. Thus, intranasal administration of the combination of GD3 and GM1 has the potential to slow down PD progression and rescue dysfunctional neurons. Most recently, intranasally administered deuterium-labeled GM1 was confirmed to reach the olfactory bulb, prefrontal cortex, SVZ, hippocampus (46), and midbrain (unpublished data), indicating successful delivery of GM1 to various brain regions via the intranasal route. Overall there is convincing evidence that gangliosides play a key role in the maintenance of the biological functions of neural cells and there is hope that this disease-modifying therapy may ameliorate biochemical and neurobiological deficiencies in such patients (42). In particular, treatment with GD3 and GM1 may represent a novel strategy to improve neurological deficit in neurodegenerative and mental disorders by regulating adult neurogenesis (44, 47).

## Gangliosides regulate extracellular vesicles and reduce microglia inflammatory response: focus on Huntington disease

Another neurodegenerative disease where ganglioside levels are affected is HD (2, 48), clinically characterized by motor symptoms such as chorea, bradykinesia, impaired speech, and dysphagia as well as non-motor symptoms, i.e., psychiatric and cognitive impairments. HD is inherited, affecting about 1:2000 families. It is caused by the pathological expansion of a cytosine-adenine-guanine (CAG) trinucleotide repeat in the *huntingtin* (*HTT*) gene. This mutation is translated into an expanded polyglutamine (polyQ) stretch in the mutant HTT protein and causes HTT misfolding and aggregation (49–51).

Studies show that levels of GM1 and other gangliosides were found to be reduced in postmortem HD brains (48), as well as in the striatum and cortex of HD mouse models (2). GM1 was also decreased in HD cell models and primary fibroblasts from HD patients, suggesting that ganglioside depletion occurs early in the disease process, preceding cell death and ruling out neurodegeneration and tissue remodeling as primary causes (2). A recent analysis comparing ganglioside levels in the plasma of HD carriers and healthy controls found no major differences in plasma GM1 levels. However, higher plasma levels of GM1 and GD1a correlated with better total functional capacity and independence, while higher GM2 levels were associated with better cognitive performance, suggesting a protective role of these gangliosides. Furthermore, lower plasma GM1 levels were predictive of more advanced HD stages, suggesting that neuroprotection by endogenous gangliosides might decline over the course of the disease, and that GM1 might be an important therapeutic target (52).

In HD mouse models, administration of GM1 proved to be a disease-modifying treatment by decreasing neurodegeneration as well as restoring both motor and non-motor functions (21, 22). On a molecular level, this was associated with a reduction of soluble and insoluble mHTT in the brain (21). Proteomics analysis of mouse brains following GM1 administration (unpublished) highlighted a putative new mechanism of action: gangliosides promote the formation of EVs (23). These nanosized, membrane-bound particles play a crucial role in intercellular communication (53–57) and, importantly, can pack misfolded proteins and promote their extracellular release, alleviating intracellular proteotoxic stress and promoting misfolded protein clearance (58–60). *In vitro* experiments using various cell types, including primary rat neurons and human I^3^Neurons, demonstrated that GM1 increases the number of EVs excreted by both wild-type and HD cells. At the same time, the amount of mHTT was shown to increase in the EVs and to decrease intracellularly. Similar effects were seen in cell models expressing pathogenic forms of αSyn and tau, suggesting the ability of GM1 to promote misfolded protein secretion via EVs might extend to other neurodegenerative conditions. Other complex gangliosides had similar or even stronger EV-promoting effects as GM1, while GM3, GD3 and asialo-GM1 had the opposite effect, i.e., inhibited the production of EVs. In line with this novel pharmacological effect of exogenously administered gangliosides, endogenous cellular ganglioside were proven to be crucial for EV secretion. Intracellular GM1 levels directly correlated with EV secretion, whereas pharmacological inhibition of ganglioside synthesis with an inhibitor of UDP-glucose ceramide glucosyltransferase (glucosyl-ceramide synthase) resulted in decreased EV production and mHTT secretion. Human HD fibroblasts and neuroblastoma N2a cells expressing an N-terminal fragment of mHTT exhibited lower GM1 levels than their wild-type counterparts and secreted fewer EVs, a deficit that was restored by GM1 administration (23). These data suggest that endogenous levels of gangliosides regulate EV production and influence misfolded protein secretion via EVs.

A potential concern regarding the promotion of EV-mediated mHTT export is whether this could facilitate the prion-like propagation of pathogenic proteins throughout the brain, a process implicated in other neurodegenerative diseases (61–63). However, this is unlikely, given that GM1 administration not only reduces mHTT levels but also mitigates neurodegeneration in HD mouse models (21, 22). One possible explanation is that GM1 not only increases EV production but also alters the composition and fate of secreted EVs (23). GM1 was shown to be incorporated into the membranes of EVs, which may influence their uptake and degradation by recipient cells (64–66).

Beyond its effects on EV secretion and proteostasis, GM1 also exerted significant anti-inflammatory effects *in vitro* and *in vivo*, on both wild-type and HD microglia, even after microglia activation with various stimuli that mimic the pro-inflammatory environment in neurodegenerative diseases (24, 25). These anti-inflammatory effects depended on the presence of the sialic acid residue and the lipid tail in GM1. GD3, GD1a, GD1b, and GT1b shared similar anti-inflammatory effects in *in vitro* models whereas GM3 and GQ1b displayed pro-inflammatory activity. The anti-inflammatory effects of GM1 and other gangliosides were partially reproduced by increasing endogenous ganglioside levels with a pharmacological activator of glucosyl-ceramide synthase, whereas inhibition of ganglioside biosynthesis exacerbated microglial activation in response to lipopolysaccharide (LPS) stimulation (24). Moreover, administration of GM1 corrected a defect in the development of immune tolerance that we have recently described in HD microglia (25), and decreased pro-inflammatory activation of monocytes from HD patients (Steinberg et al., in preparation). Although these studies demonstrated that GM1 administration suppresses NF-kB signaling in response to various inflammatory stimuli, thereby inhibiting the expression of pro-inflammatory cytokines, the precise molecular mechanisms underlying this effect remain to be elucidated.

Taken together, the neuroprotective and disease-modifying effects of GM1 in HD, along with the recently discovered broader role of gangliosides in proteostasis and modulation of inflammation - two key processes in HD and other misfolded protein disorders - highlight GM1 as a promising therapeutic candidate for HD and related neurodegenerative diseases.

## A reassessment of the Sygen acute traumatic spinal cord injury study highlighting the evidence of a beneficial effect for Sygen in the time to recover

The Sygen multicenter study (26, 67, 68), published in *Spine* in 2001, represents the largest randomized, double-blind, multicenter clinical trials performed in acute spinal cord injury (SCI). Building on promising results from a smaller single-center trial conducted in Maryland (69, 70), this study aimed to evaluate whether GM1 ganglioside (Sygen®) could promote neurologic recovery in patients with acute traumatic SCI. While the primary endpoint of the multicenter trial — the proportion of patients demonstrating “marked recovery (MR)” at 26 weeks — was not met, the study yielded a number of important secondary observations. In the Sygen multicenter study, 430 patients received SYGEN and 330 patients received placebo, with 111 MR patients in the placebo group and 144 in the SYGEN group.

**Figure 3:**
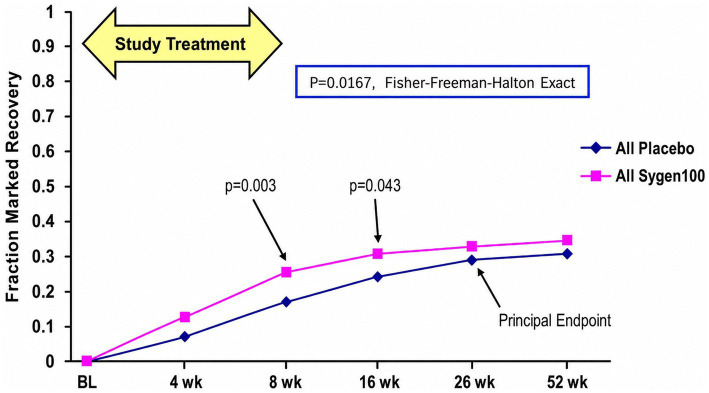
Marked recovery by visit in group of all patients. Marked recovery was defined as an improvement of 2 grades from baseline according to the AIS/Benzel classification. At the end of the 8-week treatment period, the proportions of major improvers were significantly different (*p* = 0.003). At week 16, the difference remained significant (*p* = 0.043). The protocol-specified principal endpoint at week 26 was not statistically significant. At the end of treatment, 25% of Sygen-treated patients had attained major improvement; this was not observed in the placebo group until week 16, implying that major recovery occurred approximately 2 months earlier in the Sygen group. Figures 4–6 show these recovery patterns according to baseline ASIA impairment grade.

**Figure 4:**
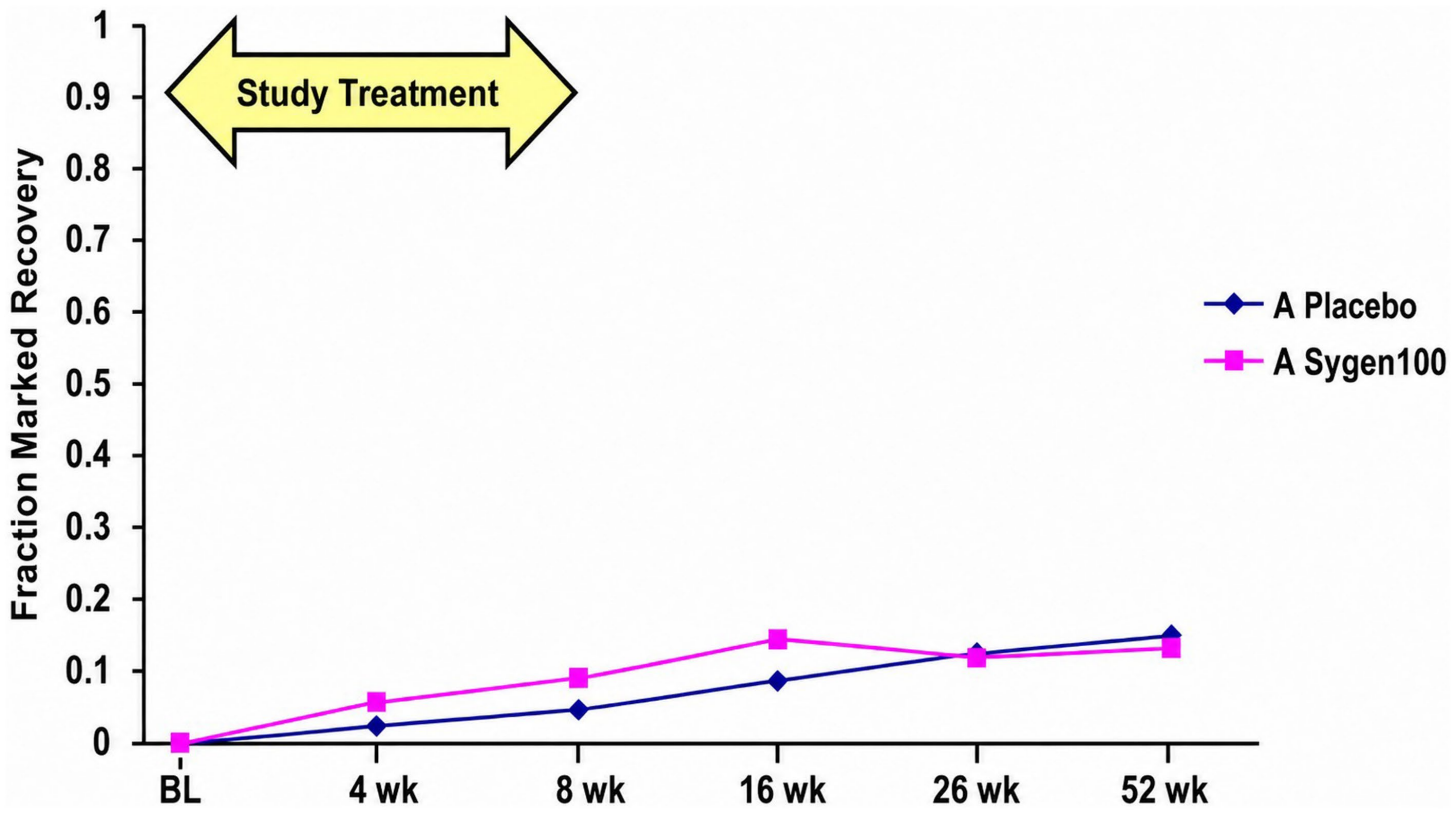
Marked recovery by visit in patients with baseline AIS grade A injury. Marked recovery was defined as an improvement of 2 grades from baseline according to the AIS/Benzel classification. Note the minimal and non-significant separation between the Sygen and placebo groups at weeks 8 and 16, favoring the drug-treated group.

**Figure 5:**
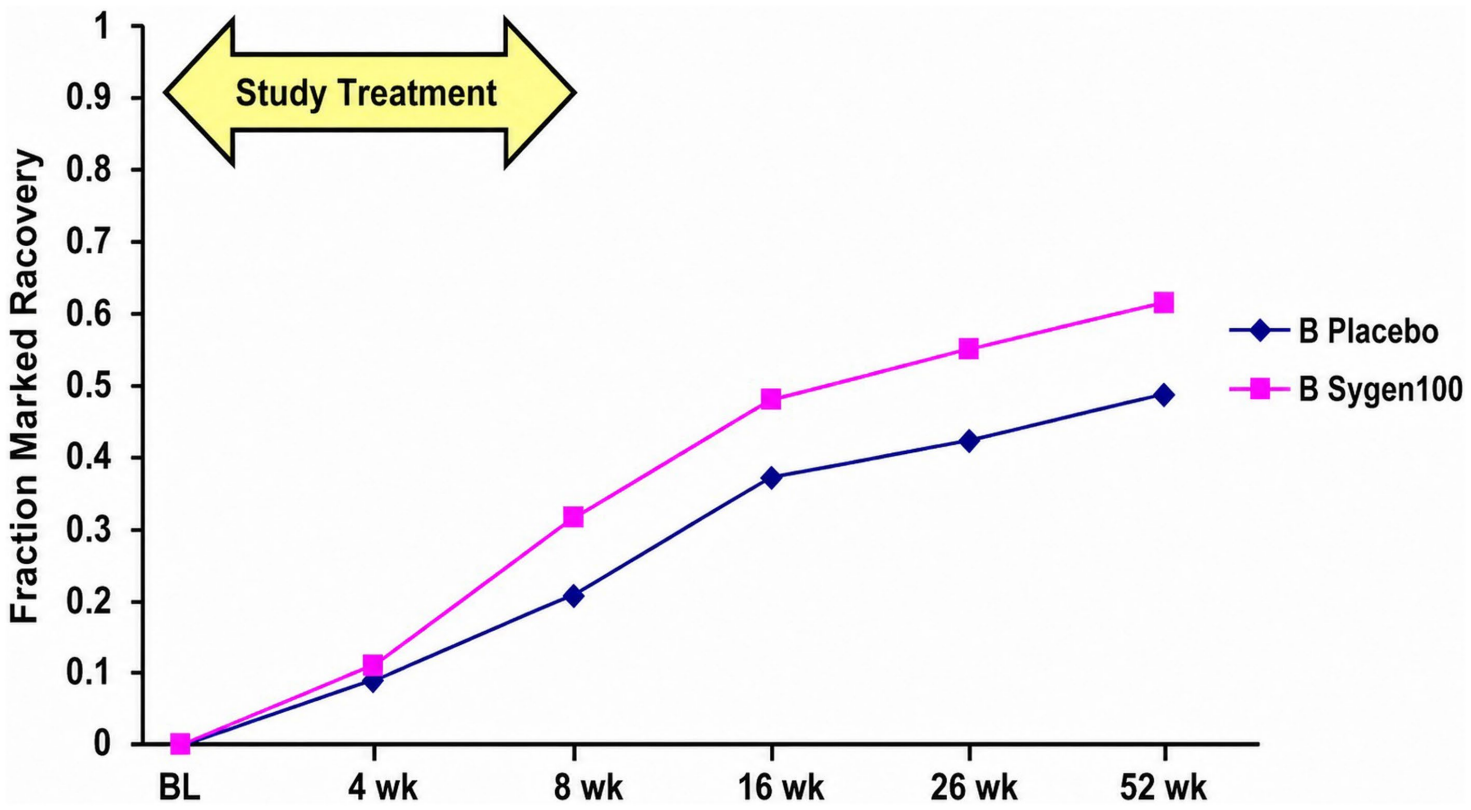
Marked recovery by visit in patients with baseline AIS grade B injury. Marked recovery was defined as an improvement of 2 grades from baseline according to the AIS/Benzel classification. Recovery occurred earlier and was greater in the Sygen group. At week 26, the Sygen group had a 55% major improvement rate, representing a greater than 30% increase over the placebo rate.

**Figure 6:**
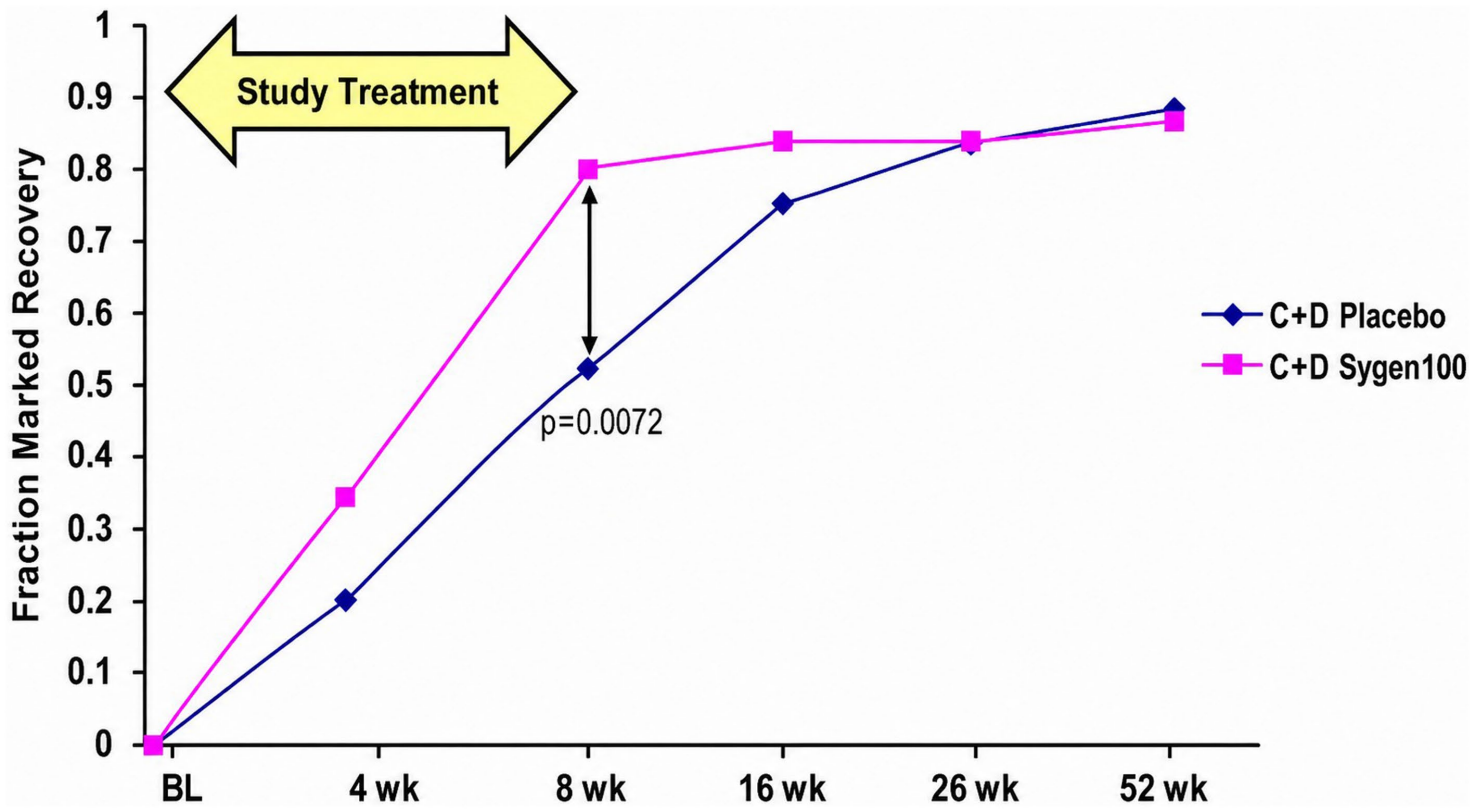
Marked recovery by visit in patients with baseline AIS grades C-D injury. Marked recovery was defined as an improvement of 2 grades from baseline according to the AIS/Benzel classification. At the end of the study treatment period, the Sygen group had a significantly greater proportion of major improvers than the placebo group (*p* = 0.0072). This significance was lost later because of a ceiling effect, with both groups reaching an 86% major improvement rate at week 26. Note again the earlier recovery in the Sygen-treated group.

Among these, are the results depicted in one of the original 2001 study articles (26), now redrawn and numbered as Figure 3. Additionally, the subgroup analysis of baseline ASIA Impairment Scale (AIS, for AIS grades see below, paragraph “Marked Recovery by Baseline Severity Groups, for definitions) in one of the original 2001 study articles (67), are now redrawn as separate baseline AIS grade in separate graphs and renumbered as Figures 4–6. These four figures stand out as particularly illustrative, providing insights into the time course of recovery and the effects of baseline AIS injury severity.

This chapter provides a reassessment of the results of the studies ^64,65^ focusing on specific endpoints-based on the clear definition of concept of “marked recovery.” In this trial, marked recovery was defined as at least a two-grade equivalent improvement from baseline ASIA grade (71) when assessed by the Modified Benzel Classification system (67), which emphasizes functional parameters such as ambulation. This threshold was chosen because it reflects a clinically meaningful degree of improvement that is directly relevant to patient independence and quality of life.

Figure 3, presents recovery over time, comparing Sygen-treated patients against placebo across all severity groups combined. At the prospectively defined primary endpoint — 26 weeks post-injury — there was no statistically significant difference between groups. This constituted the main “negative” result of the trial. However, the figure reveals a subtler and clinically important narrative: Sygen-treated patients tended to recover earlier, with an observable separation of recovery curves beginning as early as four weeks and becoming pronounced by eight weeks, the end of the 56-day treatment period. At the end of the 8 week treatment period the proportions of major improvers are statistically significantly different, *p* = 0.003. At week 16 the *p* value is 0.043.

At 8 and 16 weeks, the difference between Sygen and placebo was significant, suggesting that Sygen accelerated the attainment of marked recovery even if, by 26 weeks, placebo patients partially “caught up.” This time to recovery enhancement is compatible with a drug mechanism of action that accelerates the natural healing of the damaged spinal cord injury tissue.

The clinical implication of these findings is twofold. First, although the long-term endpoint did not demonstrate superiority, earlier recovery carries substantial functional and psychosocial advantages. Patients who achieve milestones sooner experience fewer complications, reduced dependency, and potentially greater participation in rehabilitation, which may themselves reinforce recovery. Second, the discrepancy between early and late outcomes underscores the possibility of a “ceiling effect”: as time progresses, the natural recovery trajectory of SCI patients may obscure early benefits conferred by pharmacologic intervention. This is particularly problematic in mild-to-moderate injuries, where spontaneous recovery rates are already high.

Results presented in Figure 3 thus suggest that the trial’s choice of 26 weeks as the sole primary endpoint may have underestimated the therapeutic impact of Sygen. Had the primary analysis focused on the eight-week mark, during active treatment, the results would have been positive.

The recovery patterns have been further dissected by stratifying patients according to initial American Spinal Injury Association (AIS) severity grades (Figures 4–6): In group A that consists in patients with complete injuries (Figure 4), the patients had the poorest prognosis. The marked recovery rate was very low across both Sygen and placebo arms. Although there was a slight trend favoring Sygen at weeks 8 and 16, the curves remained close together, reflecting the limited capacity for functional restitution in complete SCI. In group B that consists in patients with sensory incomplete injuries (Figure 5), Sygen showed a clear advantage. By week 26, approximately 55% of Sygen-treated patients had attained marked recovery, compared with a significantly smaller proportion in the placebo group — representing more than a 30% relative gain. Note the earlier and enhanced recovery of the Sygen group in the B’s. Importantly, the separation between curves emerged early and persisted, highlighting a sustained treatment effect in this subgroup. In groups C-D that consists in patients with motor incomplete injuries (Figure 6), the patients in these categories had the highest baseline potential for recovery. In this group, Sygen-treated patients initially recovered faster, with significantly higher rates of marked recovery at weeks 4, 8 (*p* = 0.0072), and 16. However, by week 26, placebo patients had largely caught up, with both groups converging around 85–90% recovery by 52 weeks. This convergence illustrates the “ceiling effect”: in milder injuries, recovery is so frequent that detecting a drug effect becomes statistically difficult over longer time horizons. Note again the earlier recovery in the Sygen treated group.

The stratified results emphasize that injury severity is a critical determinant of treatment responsiveness. The minimal impact in Group A patients highlights the biological limitations of therapeutic interventions in complete injuries, where neuronal loss is extensive and regenerative capacity is minimal. By contrast, the substantial benefit in Group B patients suggests that Sygen can augment intrinsic recovery mechanisms when some degree of preserved function remains, possibly by enhancing remyelination, neurite sprouting, or synaptic plasticity.

In Groups C–D, the drug’s apparent role was to accelerate rather than increase the likelihood of recovery. Clinically, faster recovery is not trivial: it may reduce hospital stays, lower costs, and improve early independence, even if final outcomes converge. However, from a regulatory perspective, acceleration alone may be insufficient to meet strict primary endpoints unless predefined accordingly.

The heterogeneity across severity groups also highlights the importance of careful trial design. Future studies might enrich for Group B-type patients, who appear most responsive, thereby maximizing the chance of detecting meaningful effects.

The data reviewed call for the following points for discussion: (i) Sygen accelerates recovery: Across all patients, the drug shifted recovery earlier, with significant differences at the end of the dosing period (week 8). The mechanism of action for a drug that accelerates recovery but has the same fraction of long-term recovery is that the drug enhances the speed of untreated natural recovery. The faster neurological recovery is clinically beneficial, as it results in a shorter rehabilitation period which achieves the same recovery foals; costs and disability related events are also both decreased. (ii) Sygen benefits certain subgroups more strongly: Group B patients with sustained differences persisting at 26 weeks. (iii) Ceiling effects obscure long-term benefits: In milder injuries (Groups C–D), the high rates of spontaneous recovery made it difficult to sustain differences by week 26, even though early recovery was accelerated. (iv) Complete injuries remain refractory: Group A patients showed little benefit, reminding us of the biological barriers to meaningful functional recovery in anatomically complete lesions.

Taken together, these results suggest that the negative primary outcome of the trial does not equate to absence of drug efficacy. Instead, they highlight the importance of selecting appropriate endpoints, patient populations, and follow-up durations to detect clinically meaningful effects. The rigid adherence to a single endpoint at 26 weeks obscured the positive signal apparent at earlier time points and in key subgroups.

Several lessons useful for designing future studies can be drawn from reassessment of the above described results from the Sygen Study: (i) Endpoint Selection: The decision to evaluate only the 26-week marked recovery rate as the primary endpoint underestimated Sygen’s impact. Future trials should focus on end of drug administration period and time to Marked Recovery as a measure to speed of recovery. (ii) Patient Stratification: the robust effect in Group B patients underscores the need for tailored inclusion criteria. Rather than recruiting across all severities, focusing on subgroups with preserved neurological substrates may maximize the likelihood of demonstrating benefit. (iii) Pathophysiological Heterogeneity: the interaction between baseline AIS grades and drug efficacy highlights that SCI is not a uniform condition. Stratifying by baseline AIS grades is critical for discovering and interpreting therapeutic responses. (iv) Clinical Relevance of Early Recovery: even if long-term outcomes converge, earlier recovery can dramatically influence patient lives. Mobility, independence, and reduced complications during the first months after injury may yield lasting quality-of-life improvements that should not be discounted. (v) Regulatory and Statistical Considerations: the trial illustrates the tension between rigid statistical frameworks and clinical realities. While the primary endpoint was negative, secondary analyses provide compelling evidence of efficacy in subgroups. This underscores the importance of adaptive designs and careful selection of outcome measures in future trials.

The Sygen multicenter trial is often summarized as a “negative” study because its predefined primary endpoint at 26 weeks did not show a statistically significant benefit of GM1 ganglioside over placebo. However, the detailed results depicted in Figures 3–6 tell a more complex story. These figures demonstrate that Sygen accelerated recovery both overall and in each baseline AIS subgroup which was particularly evident at the end of dosing; that it conferred substantial advantages in sensory-incomplete (Group B) patients.

The trial thus provides both evidence of potential therapeutic benefit and lessons in trial design. It highlights the challenges of studying heterogeneous conditions like SCI, the pitfalls of relying on a single late endpoint, and the importance of both a speed to recovery analysis and a AIS baseline subgroup analyses. While Sygen did not become a standard therapy, its study advanced the field, shaping how future interventions are conceptualized, tested, and interpreted.

## Role of GM1 in the current treatment of spinal cord injury

A country with a particularly high incidence of SCI appears to be Brazil (72). Accordingly, a more recent single-center, placebo-controlled trial was undertaken at the University Hospital in Sao Paolo to confirm whether SCI patients may benefit from GM1 rather than from methylprednisolone treatment (73). Eligible patients had to be adults between 18 and 50 years of age and to be admitted to hospital between 8 and 72 h from a closed injury of the spine from C4 to T10. The injury had to result in neurological deficit not yet to be treated with methylprednisolone. Patients were randomly assigned to treatment with either placebo or GM1 i.v., the latter at 200 mg in primary care and thereafter at 100 mg/day for 30 days. Patients were assessed for sensory (touch and pain) and motor symptoms using a standardized neurological ASIA/ISCOS score after 6 weeks, 6, 12, and 24 months. Thirty consecutive patients were enrolled and randomized to treatment groups, which showed no significant differences in demographics or injury characteristics at baseline. At all times, GM1 treated patients had higher scores for sensitivity to pain and touch as compared to placebo patients, but no benefit in motor index over 2 years, as reported by an earlier study (74). In the same hospital a larger cohort of 150 patients revealed no side effects and indicated GM1 to be safe (75).

Of the two pharmacological treatments approved for clinical use, i.e., corticosteroids and GM1, the deleterious side effects reported for massive doses of methylprednisolone made centers abstain from routinely using it for SCI. Thus, GM1 at a loading dose of 300 mg followed by 100 mg once daily for 30 days, via i.v. or i.m. injection appears currently to be the only recommendable pharmacological treatment option for patients with neurological deficits due to SCI (76).

## Conclusion

Gangliosides emerge as key regulators of CNS function, exerting coordinated effects on neuronal metabolism, plasticity, and survival. The evidence discussed highlights the central role of GM1 in modulating astrocyte–neuron interactions, promoting neurogenesis, and regulating processes such as proteostasis and inflammation. These diverse yet interconnected mechanisms underscore the importance of ganglioside homeostasis for maintaining neural integrity across physiological and pathological conditions.

Importantly, the convergence of findings from cellular, animal, and clinical studies supports the therapeutic potential of gangliosides in a wide spectrum of neurological disorders. Their ability to act on multiple cellular targets and pathways positions them as promising candidates for disease-modifying strategies, particularly in conditions characterized by metabolic dysfunction, neurodegeneration, or impaired repair processes.

While significant progress has been made, further research is required to better define the molecular mechanisms underlying ganglioside action, optimize modes of administration, and identify patient populations most likely to benefit. Continued investigation along these lines will be essential to fully realize the clinical potential of ganglioside-based therapies in neurology.
